# Chorioamnionitis and neonatal sepsis due to extended‐spectrum beta‐lactamase‐producing *Escherichia coli* infection: a case report

**DOI:** 10.1002/ccr3.5078

**Published:** 2021-11-22

**Authors:** Saheed Akinola Shittu, Sufia Athar, Adila Shaukat, Lolwa Alansari

**Affiliations:** ^1^ Department of Obstetrics and Gynaecology Al Wakra Hospital Hamad Medical Corporation Doha Qatar; ^2^ Infectious Disease Unit Division of Medicine Al Wakra Hospital Hamad Medical Corporation Doha Qatar

**Keywords:** chorioamnionitis, *E. coli*, extended‐spectrum beta‐lactamase, neonatal sepsis, wound infection

## Abstract

Chorioamnionitis is an acute inflammation of the membranes and chorion of the placenta typically due to ascending polymicrobial infection in the setting of membrane rupture. It is a common complication of pregnancy associated with significant maternal, perinatal, and long‐term adverse outcomes. We present a case of placental infection leading to preterm delivery, severe neonatal sepsis, maternal wound infection, postnatal readmission, and prolonged hospital stay. This virulent infection was caused by multidrug–resistant extended‐spectrum beta‐lactamase (ESBL)‐producing *Escherichia Coli* (*E. Coli*), which represent a major worldwide threat according to the Centre for Disease Control and Prevention (CDC). It was managed with appropriate antibiotic therapy, patient‐centered approach, and multidisciplinary team involvement that led to favourable maternal and neonatal outcome.

## Graphical abstract

Chorioamnionitis is a fetomaternal condition that can be caused by ESBL‐producing *E. coli* which pose challenging infection control issues and are a serious global threat. Screening strategies to monitor them could be useful in women from endemic areas to prevent perinatal transmission and introduction of multiresistant strains to the maternity wards.

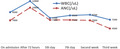

## 1 INTRODUCTION

Chorioamnionitis is a common infection of pregnancy, typically occurring in the setting of prolonged prelabor rupture of membranes (PROM) or labor. It complicates 1%–4% of all births in the United States.^1^ However, its frequency varies significantly with gestational age, specific risk factors, and diagnostic criteria. It may be diagnosed clinically, microbiologically, or by histopathological examination of the placenta and umbilical cord.^2^, ^3^

Associated risk factors include obesity, anaemia, diabetes, prolonged PROM, prolonged labor, nulliparity, African American ethnicity, multiple vaginal examinations, meconium‐stained liquor, smoking and drug abuse, epidural anaesthesia, compromised immunity and colonisation with GBS, bacterial vaginosis, sexually transmissible genital infection, and vaginal colonisation with ureaplasma.^2^, ^3^, ^4^

The main preventative strategy is administration of antibiotics to women with preterm PROM, which reduces the incidence of clinical chorioamnionitis, prolongs the time to delivery, and improves neonatal outcomes. Optimal management of clinical chorioamnionitis includes antibiotic therapy and delivery of the infected products of conception.^5^, ^6^, ^7^

We present a case of clinical chorioamnionitis associated with fetal compromise, neonatal sepsis, and severe maternal wound infection caused by multidrug–resistant ESBL‐producing *E. coli* and the challenges associated with its management.

## 2 CASE PRESENTATION

A 33‐year‐old Indian lady, G3P1 reported to the emergency department at 33 weeks with history of passing clear fluid vaginally for 1 hour. She had no associated fever, urinary symptoms, or vaginal discharge. Her previous delivery was by Caesarean section (CS) in 2014. She was on metformin for gestational diabetes diagnosed at 28 weeks.

On examination, she was clinically stable (pulse rate (PR)‐76/min, regular, blood pressure (BP)‐100/60 mmHg, and oral temperature 36.4°C). Systemic examination was unremarkable. Abdominal examination revealed fundal height of 32 cm, with normal uterine tone and regular fetal heart rate. The admission cardiotocography (CTG) was normal. Speculum examination revealed clear amniotic fluid leakage. Routine laboratory tests were sent. Antenatal steroids were offered, blood sugar monitoring with diet control was advised and sliding dose of insulin Injection was initiated. Oral Erythromycin was started for GBS prophylaxis. The patient and her family were counselled in detail regarding the conservative management and risks of chorioamnionitis and prematurity.

Her admission laboratory test results were normal (Hemoglobin‐11.6 g/dl, blood group O positive, white blood cell count (WBC) was 9.5 × 10^3^/µl, absolute neutrophil count (ANC) −7 × 10^3^/µl, and C‐reactive protein (CRP) −7 mg/L). Ultrasound scan revealed a small for date fetus with growth corresponding to <7th centile with oligohydramnios and normal uterine artery Dopplers. She was clinically stable in the first 48 h and twice‐daily CTG remained normal. However, she later developed tachycardia (PR‐122/min) and increased WBC (12.9 × 10^3^/µl) and ANC (10.9 × 10^3^/µl) were noted in her blood count but she was afebrile. CTG done at this time revealed baseline tachycardia of 180 bpm, reduced variability of 3–5 bpm; no acceleration, but had unprovoked recurrent decelerations followed by prolonged deceleration for more than 3 minutes.

**FIGURE 1:**
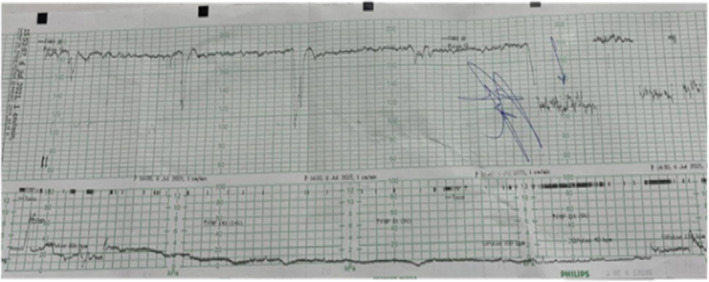
Cardiotocography (CTG) prior the decision for delivery

In view of pathological CTG findings shown in Figure 1, she had emergency CS for fetal compromise as the cervix was only 1‐cm dilated. Routine prophylactic antibiotics, cefazolin and azithromycin, were administered prior to surgery as she was afebrile at that time. The baby was male weighing 1570 g, and Apgar scores were 9^1^,10^5^. The liquor was meconium stained and foul smelling. The arterial pH and the venous pH were 7.30 and 7.36, respectively. The base excess was −0.7 and −0.6 mmol/L for the arterial and venous sample. Placental tissue was sent for culture and sensitivity as clinical diagnosis of chorioamnionitis was made and broad‐spectrum antibiotics (ceftriaxone and metronidazole) were started.

**FIGURE 2:**
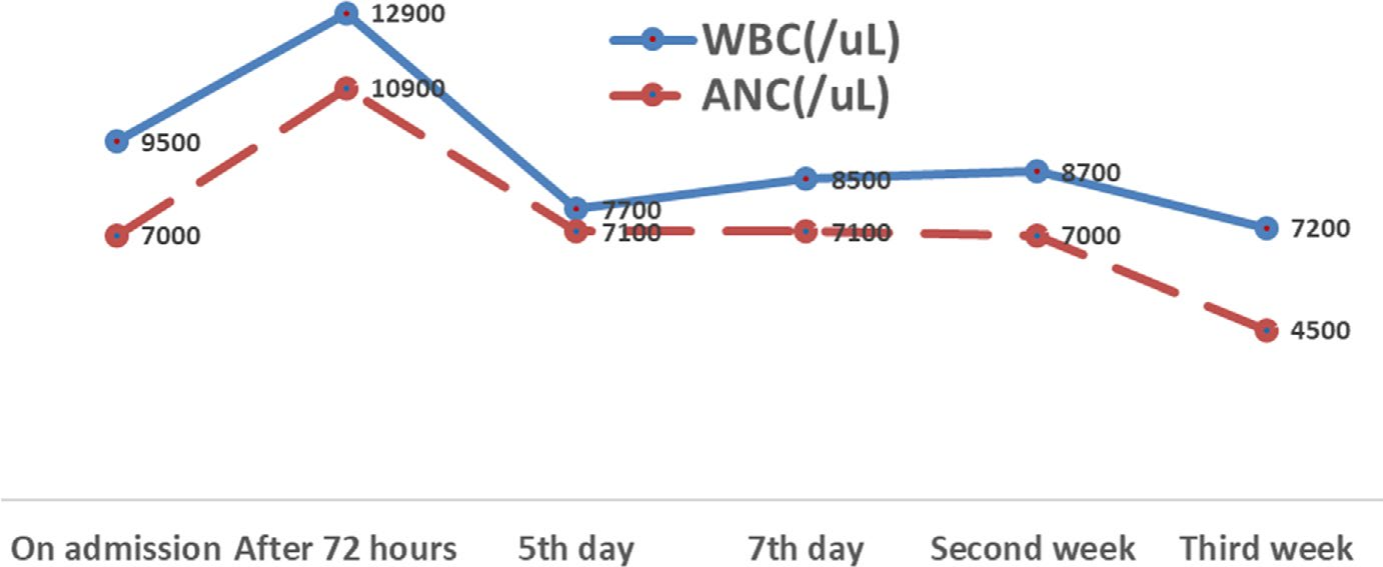
White blood count (WBC) and absolute neutrophil count (ANC) of the patient from admission

**FIGURE 3:**
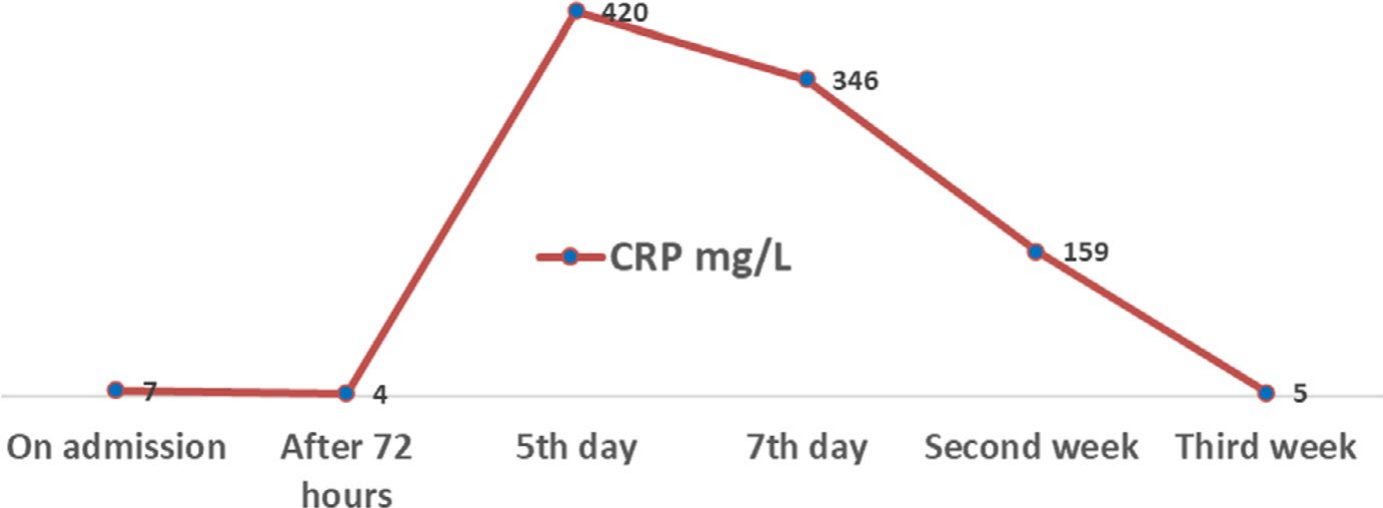
C‐reactive protein (CRP) levels of the patient from admission

However, she had persistent tachycardia with increasing CRP levels (420 mg/L). The culture result of placental tissue and high‐vaginal swab (HVS) confirmed profuse growth of ESBL‐producing *E. coli*. confirming microbiological chorioamnionitis. Infectious disease team was involved and IV (intravenous) Ertapenem was commenced based on sensitivity profile of the result of culture. She remained clinically stable for 72 h, and WBC counts and CRP were showing a decreasing trend (Figures 2 and 3). She was discharged home on IV Ertapenem to be taken on outpatient basis. However, on post‐operative day 6, she was readmitted because of wound infection and oral temperature was 38.2℃. Wound swab culture also grew ESBL‐producing *E. coli* and antibiotic was changed to IV Meropenem. Wound care team was involved.

After instituting Meropenem, she recovered well, and the blood counts and CRP were reduced to near normal. Meropenem was continued for 7 days. She was discharged home in clinically stable condition.

The baby born vigorous was initially on continuous positive airway pressure, but within few hours of birth developed signs of sepsis (PR‐186 bpm, BP‐44/30 mmHg, temperature 37.9°C, and SpO2 dropped to 93%) with respiratory distress syndrome and was intubated and required inotropes (dopamine infusion). Broad‐spectrum antibiotics (Amikacin and Ampicillin) were given. Blood culture confirmed growth of gram‐negative ESBL‐producing *E. coli* within 9 hours of birth, so Meropenem was added, and Ampicillin was discontinued. Fetal Echocardiography showed dilated inferior vena cava with evidence of pulmonary hypertension.

Baby developed high fever (39.5°C) on second day of birth, requiring paracetamol. Total parenteral nutrition (TPN) was initiated. Laboratory results were suggestive of leucopenia (WBC 4.5 × 10^3^/µl), low platelet count (96 × 10^3^/µl), high CRP (56 mg/L), high serum Urea (9.7mmol/L), high serum creatinine (76 umol/L), and high total bilirubin levels(142.3µmol/L). Due to persistent hypotension, higher doses of Dopamine infusion were given. On day 4, hemoglobin level dropped from 19 g/dl to 15 g/dl so skull X‐ray and brain ultrasound were performed and intracranial bleeding was excluded. In addition to sepsis, neonatal jaundice was noted, and ABO incompatibility was diagnosed. Lumber puncture was performed, which revealed leukocytosis (216/µl and raised RBCs (11/µl). Though culture showed no growth, he was treated for meningitis.

Gradually in 10 days, the baby improved clinically. TPN was continued for 12 days and then oral feeding was commenced. Neonatal Jaundiced settled without the need for phototherapy. Antibiotic (Meropenem) was given for four weeks. Baby was discharged on 28th day of admission.

## 3 DISCUSSION

The patient was afebrile earlier unlike most cases of clinical chorioamnionitis^1^, ^3^, ^4^; hence, the diagnosis of clinical chorioamnionitis was confirmed during the CS by foul‐smelling liquor and broad‐spectrum antibiotics were commenced post‐operatively. The risk factors were preterm PROM and gestational diabetes. This confirmed that knowing the risk factors can add to the diagnostic accuracy, as in all situation treatment needs to be initiated based on clinical diagnosis.^2^, ^4^

The definition of chorioamnionitis varies according to the key diagnostic criteria, which can be given as follows: clinical, based on the presence of typical findings such as maternal fever, tachycardia and foul‐smelling liquor, or vaginal discharge; microbiological, based on the culture of microbes from amniotic fluid obtained by amniocentesis or placenta; and histological, based on microscopic evidence of maternal or fetal inflammatory response on examination of placenta or membranes. Histological chorioamnionitis is a more common diagnosis as it includes both subclinical and clinical chorioamnionitis. Funisitis occurs when infection/inflammation extends to the umbilical cord.^1^, ^2^, ^3^, ^4^

The clinical diagnosis of chorioamnionitis in women with preterm PROM should be made by use of a combination of clinical assessment (including CTG) and tests discussed below (WBC, CRP, etc.). It is important that the results of the clinical assessment or any of the tests are consistent with each other, or else observation and repetition of the tests should be considered.^7^

Our patient's CTG was pathological as shown in Figure 1 and suggestive of fetal compromise, but the cord pH and base excess were normal. The role of CTG in the diagnosis of chorioamnionitis is uncertain as it is a test widely used for fetal hypoxia; but chorioamnionitis causes nonhypoxic fetal compromise.^6^ Be that as it may, CTG abnormalities are frequent among patients with chorioamnionitis. Baseline fetal tachycardia more than 160 bpm is a time‐honored early sign of chorioamnionitis. Other CTG features such as reduced variability, deceleration, lack of accelerations, and lack of cycling with or without features of tachysystole or hyperstimulation may be markers of chorioamnionitis that may be associated with adverse perinatal outcome as seen in our patient.^5^, ^6^

When chorioamnionitis is suspected in the absence typical clinical signs (subclinical chorioamnionitis), biochemical, serum or amniotic fluid tests may be used to make diagnosis. Amniocentesis for amniotic fluid culture is the best method for diagnosis of subclinical chorioamnionitis in preterm gestations but it is considered invasive. Other tests that can be obtained rapidly include WBC, CRP level, leukocyte esterase level, gram stain and glucose concentration, and cytokine level (IL‐6).^1^, ^5^, ^6^, ^7^, ^8^

Blood culture was not done in our patient, as she initially did not have fever. Ideally, this should be done prior to administration of antibiotics which should be given within an hour of recognition of severe sepsis and sepsis bundle protocol should be implemented. Critical care outreach team should be contacted if indicated.^8^

The definitive diagnosis of chorioamnionitis in this case was based on microbiological test of placenta culture growing ESBL organism. Unfortunately, placental histology was not obtained. Chorioamnionitis confirmed on histopathology is associated with an increase in CS rate due to fetal heart rate changes, increased risk of wound infection in mothers, and increased admission of the babies to neonatal intensive care units as seen in this case. More importantly, it is a reliable indicator of infection even when the infection is not clinically apparent.^12^

The maternal complications of chorioamnionitis are postpartum hemorrhage, endometritis, wound infection, sepsis, and death. The neonatal complications include premature birth, stillbirth, neonatal sepsis, chronic lung disease and brain injury causing cerebral palsy, and other neuro‐developmental disabilities.^1^, ^5^, ^6^

Route of infection is ascending infection in 96% of cases. The remainder is from haematogenous spread due to maternal septicemia^13^, iatrogenic from procedures such as amniocentesis and chorionic villous sampling. Infection from the peritoneum via the fallopian tubes has been postulated.^1^, ^2^

The most common organisms identified in pregnant women dying from sepsis are genital mycoplasmas, Lancefield group A beta hemolytic streptococcus and *E. coli*.^10^ While GBS and *E. coli* are the two most common pathogenic organisms for early‐onset neonatal sepsis, ESBL‐producing multidrug–resistant *E. coli* has emerged as the major pathogen responsible particularly in preterm infants.^10^, ^11^

*Escherichia coli* belongs to the family of Enterobacteriaceae—a large order of different types of bacteria that commonly cause infections both in healthcare settings and in communities. To survive the effects of antibiotics, some Enterobacteriaceae produce ESBL enzyme that destroys and renders ineffective commonly used antibiotics such as penicillins and cephalosporins, but not cephamycin and carbapenems. This resistance means fewer antibiotic options are available to treat ESBL‐producing bacterial infections and even more common infections caused by such organisms may require more complex treatment requiring prolonged hospitalisation and intravenous carbapenem antibiotics—a class of highly effective antibiotic agents commonly reserved for the treatment of severe or high‐risk multidrug–resistant bacterial infections.^9^, ^11^ Therefore, appropriate and justified use of these antibiotics is necessary to decrease the risk of emergence of resistance.

Our patient's cultured organism from placental tissue and the wound culture was resistant to commonly used antibiotics and was sensitive only to Meropenem, Ertapenem and Gentamicin. Resistance that renders these antibiotics ineffective are on the rise too. The more we rely on these antibiotics, the greater the risk of spreading resistance to them. This mandates the practice of sending appropriate specimens for culture before initiating antimicrobial therapy and the implementation of stewardship programs to regulate the appropriate use of antimicrobials as well as, robust infection control practices that help to prevent cross transmission of infection.^15^

Extended‐Spectrum Beta‐Lactamase ‐producing organisms were first found in Europe, and the earliest cases were identified in the USA in 1988.^14^ A pooled prevalence of faecal colonisation of 14% globally was estimated by a systematic review,^16^ and Villa HE et al.^14^ in Argentina quoted 5.4% of pregnant women in their study had ESBL‐producing *E. coli* vaginal colonisation revealing that colonisation with resistant *E. coli* is significant in pregnancy. A higher prevalence of 22% was reported in South‐Asia^16^ where our patient came from.

According to CDC, ESBL‐producing *E*. *coli* pose challenging infection control issues and are a serious global threat that requires prompt and sustained action.^16^ Appropriate use of antibiotic therapy under the guidance of infectious disease team is imperative. Prophylactic use of antibiotics during conservative management of PROM and prompt delivery when indicated will help reduce the sequelae of infection. Screening strategies designed to monitor for ESBL‐producing *E. coli* could be useful in women from endemic areas to prevent perinatal transmission and the introduction of multiresistant strains to the maternity ward.

## CONFLICT OF INTEREST

The authors have no conflict of interest to disclose.

## AUTHOR CONTRIBUTION

SAS and SA conceived the idea and reviewed literature. SAS, SA, AS, and LA contributed to care of patient and her neonate and drafting of the manuscript. All authors read and approved the final version.

## ETHICAL APPROVAL

Institutional ethics committee approval was obtained for this case report, and written informed consent was obtained from all participants.

## CONSENT

Written informed consent was obtained from the patient/parents for their anonymized information to be published in this article. Documentation of the written consent will be provided to the journal upon request.

## Data Availability

All data containing relevant information to support the study findings are provided in the manuscript.
